# Unravelling the Regions of Mutant F508del-CFTR More Susceptible to the Action of Four Cystic Fibrosis Correctors

**DOI:** 10.3390/ijms20215463

**Published:** 2019-11-01

**Authors:** Giulia Amico, Chiara Brandas, Oscar Moran, Debora Baroni

**Affiliations:** Istituto di Biofisica, Consiglio Nazionale delle Ricerche, 16149 Genova, Italy; giulia.amico87@gmail.com (G.A.); chiara.brandas01@universitadipavia.it (C.B.); oscar.moran@cnr.it (O.M.)

**Keywords:** cystic fibrosis transmembrane conductance regulator (CFTR), cystic fibrosis, F508del-CFTR, CFTR-correctors, CFTR halves, transmembrane-nucleotide binding domains

## Abstract

Cystic fibrosis (CF) is a genetic disease associated with the defective function of the cystic fibrosis transmembrane conductance regulator (CFTR) protein that causes obstructive disease and chronic bacterial infections in airway epithelia. The most prevalent CF-causing mutation, the deletion of phenylalanine at position 508 (F508del), leads to CFTR misfolding, trafficking defects and premature degradation. A number of correctors that are able to partially rescue F508del-CFTR processing defects have been identified. Clinical trials have demonstrated that, unfortunately, mono-therapy with the best correctors identified to date does not ameliorate lung function or sweat chloride concentration in homozygous F508del patients. Understanding the mechanisms exerted by currently available correctors to increase mutant F508del-CFTR expression is essential for the development of new CF-therapeutics. We investigated the activity of correctors on the mutant F508del and wild type (WT) CFTR to identify the protein domains whose expression is mostly affected by the action of correctors, and we investigated their mechanisms of action. We found that the four correctors under study, lumacaftor (VX809), the quinazoline derivative VX325, the bithiazole compound corr4a, and the new molecule tezacaftor (VX661), do not influence either the total expression or the maturation of the WT-CFTR transiently expressed in human embryonic kidney 293 (HEK293) cells. Contrarily, they significantly enhance the expression and the maturation of the full length F508del molecule. Three out of four correctors, VX809, VX661 and VX325, seem to specifically improve the expression and the maturation of the mutant CFTR N-half (M1N1, residues 1–633). By contrast, the CFTR C-half (M2N2, residues 837–1480) appears to be the region mainly affected by corr4a. VX809 was shown to stabilize both the WT- and F508del-CFTR N-half isoforms, while VX661 and VX325 demonstrated the ability to enhance the stability only of the mutant F508del polypeptide.

## 1. Introduction

Cystic fibrosis (CF) is a fatal autosomal recessive inherited disease caused by loss of function mutations in the CF transmembrane conductance regulator *(CFTR)* gene, which encodes a cAMP-regulated chloride and bicarbonate channel expressed at the apical membrane of epithelial cells in the airways, pancreas, testis, and other tissues [1]. There, the CFTR’s physiological role consists of regulating salt and water homoeostasis. The most common *CFTR* mutation producing CF (http.//www.genet.sickkids.on.ca) is the deletion of the codon coding for phenylalanine at residue 508 (F508del) in the CFTR protein amino acid sequence, which is present in at least one allele in approximately 90% of CF subjects [2]. The main morbidity feature due to the presence of the defective F508del-CFTR consists of a thick tenacious mucus that obstructs distal airways and sub-mucosal glands in the lungs. As a consequence of the alterations in airway surface liquid regulation and mucus consistence, the lungs are colonized by opportunistic bacteria and suffer from a rapid functional decline of respiratory function, eventually culminating in lung failure [3].

The 1480-amino acid CFTR protein shares structural and folding features with the other members of the ATP-binding cassette (ABC) transporter family. It is comprised of two homologous repeats, each consisting of six transmembrane (TMD1 and TMD2) regions followed by a cytoplasmic nucleotide binding domain (NBD1 and NBD2). The two CFTR halves are joined by a cytoplasmic regulatory (RD) domain which contains multiple consensus sequence sites for phosphorylation by protein kinases A and C [1,4]. CFTR biogenesis, folding, and trafficking to the plasma membrane are complex and multi-step processes that take place in different cellular compartments and involve several cellular components, some of which may be cell type-specific [5,6]. The F508del mutation inhibits these hierarchical processes and reduces the surface expression of the mutant CFTR [6,7]. Because of its defects, the F508del-CFTR is rapidly recognized by ubiquitination complexes that direct the protein to the endoplasmic reticulum-associated degradation pathway (ERAD) [6,8,9,10]. A little of the F508del CFTR that is successfully and properly delivered to its native destination retains some functional activity, albeit with altered gating with respect to wild type (WT) CFTR [11,12,13,14].

Since the discovery that the cell surface expression defects of F508del-CFTR and other processing mutants could be partially rescued in vitro by incubating cells that the express mutant CFTR at a low temperature (27 °C) [11], in the presence of nonspecific osmolytes such as glycerol [1,15,16], or with organic solutes such as myoinositol [17], research in CF has been oriented towards the development of a drug-mediated rescue approach. A high-throughput screening of chemical compound libraries identified several small molecules, named correctors, that are able to increase the amount of the F508del-CFTR delivered to the cell surface [18,19,20,21,22]. A problem with correctors is that in patients carrying homozygously the F508del mutation, they cause only a modest increase in the maturation of the mutant protein so that the yield of the mature CFTR is 5–10% of that of WT [23,24]. In particular, clinical trials have shown that therapy with Lumacaftor (VX809), the most effective corrector identified so far, does not significantly improve lung function, sweat chloride concentration, or the maturation of the F508del-CFTR [25]. However, a combination of VX809 with Ivacaftor (VX770, a potentiator that improves channel function) or VX770 with new proposed correctors such as tezacaftor (VX661), VX152 and VX440 has shown some improvements.

The rational development of a “second generation” of more efficient correctors is deeply limited by the lack of knowledge about their mechanism of action. While potentiators are generally believed to directly bind the mutant CFTR, improving channel gating [26], the mechanism of action of correctors is still controversial. Correctors may act as “pharmacological chaperones,” directly interacting with the F508del-CFTR itself and facilitating its folding and cellular processing [23,27,28,29,30,31,32,33,34,35,36,37,38]. Some studies have proposed that the VX809 corrector binds to the pocket formed at the NDB1/ICL4 interface [32,33,39,40], leading to the stabilization of the F508del-CFTR at the early stages of its biogenesis [41]. Other experimental studies have identified TMD1, NBD2 and NBD1 as putative targets of the VX809 [34], corr4a [35,42] and RDR1 [43] correctors, respectively. Some evidence has indicated that the VX325 corrector directly interacts with the CFTR by interacting with NBD1 [44], even if the identification of the bithiazole rescue site on the CFTR is controversial because some evidence has indicated that this compound binds to NBD2 or to the TMDs [27,34,35]. Still, it cannot be yet completely excluded that correctors may modulate the expression of the CFTR through their interaction with the protein machineries involved in the cellular quality control or with the ubiquitin proteasome-mediated degradation pathways [45,46].

Finally, it has to be noted that correctors have been divided into three different classes on the basis of their mechanism of action. VX809 and VX661 have been ascribed as class I correctors, compounds that are thought to stabilize the NBD1-TMD1/TMD2 interface; differently, corr4a, which belongs to the class II correctors, is hypothesized to restore the stability of NBD2 or its interface with other domains of the CFTR. Class III correctors facilitate NBD1 folding or impede its unfolding [27,28,29]. This mechanistic classification implies that the therapeutically strategic use of a combination of correctors targeting two or more F508del-CFTR structural defects may improve or even restore the function of the mutant CFTR, definitively improving CF patients’ symptoms and life conditions.

In this work, we used different complimentary biochemical approaches to identify the CFTR domains that are mainly involved in the rescue of the mutant F508del-CFTR to assess whether these drugs exert a different effect on the WT- and F508del-CFTR isoforms and to investigate whether tested compounds act through the same mechanisms to increase CFTR expression. Aware that the use of a heterologous expression system gives a higher outcome in the expression of the whole CFTR channel function with respect to primary cells, we intentionally chose to use transfected cells to compare the results obtained with CFTR segment constructs and the whole protein in an homogeneous cellular system, exploiting the properties of these cellular systems to conserve the functional properties of the membrane proteins [47,48,49].

We found that all assayed correctors were able to increase the functional expression of the F508del-CFTR that was heterologously expressed in highly transfectable human embryonic kidney 293 (HEK-t) cells. On the contrary, treatment with correctors did not exert any effect on both the expression and maturation of WT-CFTR transiently transfected in HEK-t cells. Then, we split the CFTR into two halves: The N-half (M1N1, residues 1–633) and the C-half (M2N2, residues 837–1480) and evaluated the effect of correctors on these regions, whose constructs were expressed either alone or together. We found that the VX809, VX661 and VX325 correctors increased the expression and the maturation of the CFTR N-half, either in its WT- and F508del- isoforms. Furthermore, the VX809, VX661 and VX325 correctors demonstrated the ability to increase the stability of the F508del-CFTR protein N-half. The corr4a corrector did not exert any effect on the expression, maturation or stability of the CFTR N-half. On the contrary, it increased the expression and the maturation of F508del-CFTR constructs where the NBD2 domain was expressed (whole F508del-CFTR, N-half F508del-CFTR + C-half) and exerted no effect on the expression of the CFTR construct lacking the NBD2 domain (ΔNBD2, residues 1–1172) either in its WT or F508del conformations. We are confident that our findings will provide an improvement to the understanding of the structural basis of the correction mechanism mediated by correctors as well as new insights for the design of new structure-based CF therapeutics.

## 2. Results

### 2.1. Effect of Correctors on the Expression of M1N1, M2N2, ∆NBD2 and CFTR mRNAs.

The relative abundance of constructs codifying for the full length WT- or F508del-CFTR, WT- or F508del-M1N1, and M2N2, WT- or F508del-∆NBD2 was evaluated in HEK-t transfected cells by qRT-PCR, using specific primer pair sets. The expression levels of the mRNA coding for the full length WT- and F508del-CFTR constructs in transfected HEK-t cells were not statistically different. Interestingly, the treatment of cells with the VX809, VX661, corr4a and VX325 correctors did not modify the CFTR mRNA expression in either in the WT-CFTR transfected cells or in the F508del-CFTR transfected cells. Data are presented in the Table S1.

The relative abundance of WT-M1N1 mRNA in the untreated transfected cells was not changed by the treatment with the VX809, VX661, corr4a and VX325 correctors. Similarly, the F508del-M1N1 mRNA yield in untreated cells was not modified by the treatment with correctors. Data are presented in the Table S2. Analogously, treatment with correctors did not change the expression level of the M2N2 mRNA in transiently transfected HEK-t cells (Table S3). When cells were co-transfected with M1N1 and M2N2, none of the examined correctors altered the relative abundance of either the WT-M1N1 of F508del-M1N1 mRNAs. In the same samples, the relative abundance of the M2N2 mRNA was not modified by the treatment with correctors. Data are shown in Table S4.

Finally, the mRNAs extracted from cells transfected with WT- or F508del-∆NBD2 had similar results in untreated cells or in cells treated with the VX809, VX661, corr4a and VX325 correctors. Data are presented in Table S5. In untransfected HEK-t cells, mRNA codifying for the full length CFTR, M1N1, M2N2, and ∆NBD2 was not detected (data not shown). We concluded that none of the correctors studied here modify the mRNA transcription of WT or F508del whole length or of any segment of the CFTR.

### 2.2. Effects of Correctors on the Functional Expression of Full Length WT- and F508del-Proteins.

**Figure 1:**
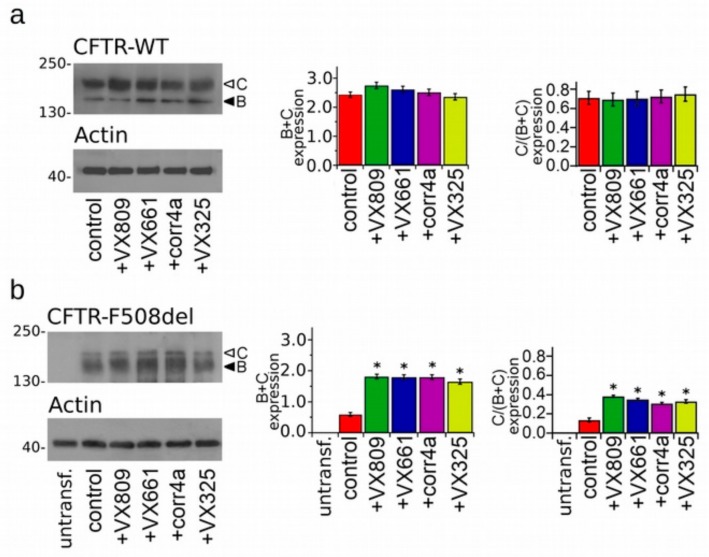
Detection of full-length cystic fibrosis transmembrane conductance regulator (CFTR) proteins. (**a**) Western blot showing the wild type (WT)-CFTR in lysates of transiently transfected human embryonic kidney 293 (HEK-t) cells treated with DMSO (control), lumacaftor (VX809), tezacaftor (VX661), corr4a, and VX325. (**b**) Expression of the phenylalanine at position 508 (F508del)-CFTR in untransfected (untransf.) and in transiently transfected HEK-t whole cell lysates treated with DMSO (control), VX809, VX661, corr4a and VX325. The expression of the housekeeper protein actin in the same samples of (**a**,**b**) is shown in the lower panels. The molecular weight of the proteins of the molecular weight marker that was run in the SDS-PAGE is indicated on the left of each blot. White and black arrowheads indicate the position of the B and C bands, respectively. Bar graphs in the middle show the quantification of the total protein expression, calculated as the sum of the B and C band. Bar graphs on the right indicate the quantification of the mature, fully glycosylated fraction of the CFTR protein, expressed as a C/(C + B) band ratio. The expression level of each band was normalized to the level of actin detected in the same samples. Data are expressed as mean ± standard error of the mean (SEM) of at least four independent experiments. Statistical comparison of the data was made by a Dunnet multiple comparison test (all groups against the control group). Asterisks indicate a statistical significance versus the control: * *p* < 0.05.

To test the effectiveness of correctors to increase the protein expression of the WT- and F508del-CFTR isoforms, we treated the WT- and F508del-CFTR transiently transfected HEK-t cells for 18 h with 5 µM VX809, 5 µM VX661, 10 µM corr4a and 5 µM VX325 or with DMSO as a control. The immunoblot analysis of the retrieved whole cell extracts is presented in Figure 1a,b. Both isoforms were detected by the monoclonal antibody MM13-4 raised against the N-terminal of the CFTR protein, as two electrophoretic bands, named B and C, of approximately 160 and 180 KDa, respectively. The B band corresponds to the core-glycosylated CFTR which resides in the endoplasmic reticulum (ER), and the C band represents the mature, fully processed CFTR that has passed through the Golgi apparatus. As expected, the prevalent form of the WT-CFTR in transfected HEK-t cells presented as the C band (the first lane of the upper panel of Figure 1a). Lysates of cells expressing the F508del-CFTR primarily showed the B band, which is consistent with the severe folding and trafficking defects caused by the mutation (first lane of the upper panel of Figure 1b). The bar graphs of Figure 1 show that the treatment of cells transfected with the full length WT-CFTR with correctors did not change either the total expression of the WT-CFTR (C + B band) or its maturation rate, which was expressed as the ratio between the expression of the mature, fully glycosylated protein (band C) and the total CFTR protein loading (B + C band). On the contrary, the treatment of F508del-CFTR transfected cells with correctors significantly enhanced both the total expression (B + C band) and the maturation of F508del-CFTR, as seen from the change in the C/(C + B) band ratio. VX809, VX661 and corr4a were almost equivalent in rescuing the expression of the F508del-CFTR. The order of the correctors’ efficacy in promoting the maturation of the mature, fully glycosylated form of F508del-CFTR was VX809 > VX661 > VX325 > corr4a.

**Figure 2:**
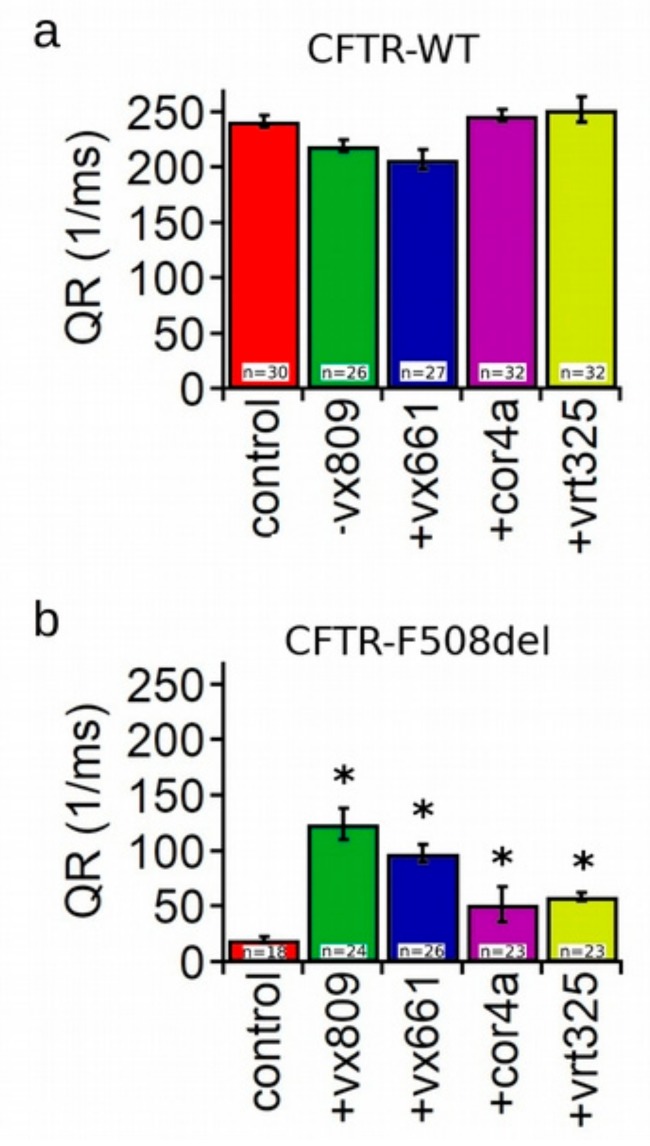
Evaluation of CFTR channel function. Channel activity is expressed as the initial fluorescence quenching rate (QR) of the yellow fluorescent protein (YFP) protein by the iodide influx in Fischer rat thyroid (FRT) cells permanently co-transfected with the WT-CFTR (**a**) and the F508del-CFTR (**b**). Cells were treated with DMSO, 5 µM VX809, 5 µM VX661, 10 µM corr4a and 5 µM VX325. Data are expressed as mean ± standard error of the mean (SEM). For each condition, the number of measurements (*n*) is indicated inside the bars of the graphs. Asterisks indicate a significant difference (* *p* < 0.05) with respect to the control, DMSO-treated samples.

To verify whether the rescue of the folding and trafficking defects of the mutant CFTR by correctors results in an increase of cell surface expression, we examined the CFTR functional anion channel activity upon forskolin activation. To achieve this goal, we evaluated the transport of iodide mediated by the WT- and F508del-CFTR proteins permanently transfected into Fischer rat thyroid (FRT) cells which expressed the iodide-sensitive yellow fluorescent protein (YFP). The iodide influx mediated by forskolin-activated CFTR channels was measured as the initial quenching rate (QR) of the YFP fluorescence. Figure 2a shows that treatment with correctors did not elicit any significant augmentation in the iodide influx in WT-CFTR FRT cells. On the contrary, the QR was significantly increased in forskolin-activated F508del-CFTR FRT cells treated with each of the correctors under analysis. In particular, there was a 4.18-fold higher increase of the iodide influx elicited in FRT cells treated with the VX809 corrector than that of the control, untreated cells. The QR in FRT-F508del cell preparations treated with correctors was VX809 > VX661 > VX325 ≥ corr4a (Figure 2b).

### 2.3. Effect of Correctors on the Expression of M1N1, M1N1 + M2N2, and ∆NBD2 Polypeptides.

**Figure 3:**
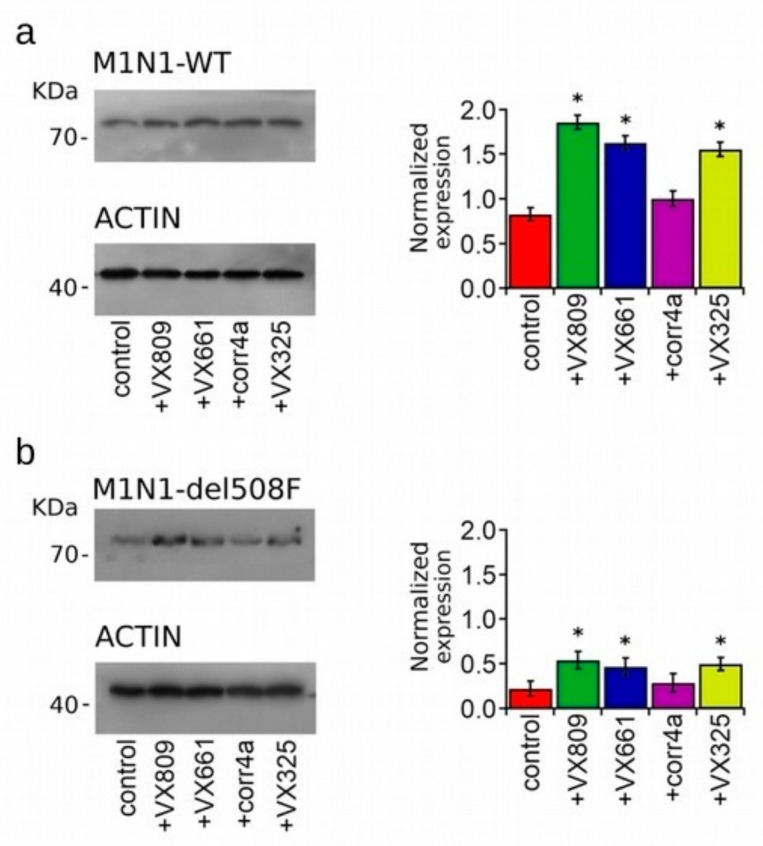
Effect of correctors on the expression of the CFTR N-half. Western blots of WT- (**a**) and F508del-M1N1 (**b**) transiently transfected in HEK-t cells treated with DMSO (control) or with 5 µM VX809, 5 µM VX661, 10 µM corr4a and 5 µM VX325. In the lower blots, the expression of actin, used as housekeeping protein, is shown. The molecular weight of the proteins of the molecular weight marker that was run in the SDS-PAGE is indicated on the left of each blot. The bar graphs on the right indicate the normalized expression level of WT- and F508del- M1N1. Data are expressed as mean ± standard error of the mean (SEM) of at least four independent experiments. The Dunnet test was used for data comparison. Asterisks indicate a statistical significance versus the control: * *p* < 0.05.

Western blot images displaying the expression of WT- and F508del-M1N1 constructs in the whole cell lysates of HEK-t transfected cells are shown in Figure 3a,b respectively. WT- and F508del-M1N1 proteins were revealed as electrophoretic bands of ~72 KDa, as expected from the predicted size of the CFTR N-half. In accordance with the sequence analysis, which did not predict any glycosylation site, WT- and F508del-M1N1 molecules were not glycosylated. The quantification of band intensity shown in the right panels of Figure 3a,b shows that the expression levels of the WT- and F508del proteins were quite different, and WT-M1N1 was 3.8-fold more expressed than F508del. Treatment with the VX809, VX661 and VX325 correctors significantly enhanced the expression of both the WT- and F508del-M1N1 proteins. However, the expression level of F508del-M1N1 after treatment with the VX809, VX661 and VX325 correctors remained significantly lower than that of the control, untreated WT isoform. Contrarily, the corr4a corrector did not increase the expression level of either WT- or F508del-M1N1 polypeptides.

Figure S1A shows a western blot image of the M2N2 polypeptide transiently expressed in HEK-t cells. Though this polypeptide comprises the two CFTR glycosylation sites located in the TMD2, it was revealed in untreated cells as only one electrophoretic band of ~90 KDa corresponding to the immature, core-glycosylated protein. The mature fully glycosylated form of the M2N2 was not detected in the untreated cells. Treatment with correctors did not promote either the appearance of the fully glycosylated form of the M2N2 polypeptide (thus confirming that the CFTR C-half does not mature unless it is co-expressed with its counterpart, the M1N1 polypeptide) [33,34], or increased the expression level of the immature form. The M3A7 antibody raised against the NDB2 domain that was used to recognize the M2N2 polypeptide detected also a band of ~73 KDa, probably corresponding to the unglycosylated form of the CFTR C-half. This band was not considered for the quantification analysis shown in the bar graph of Figure S1B.

**Figure 4:**
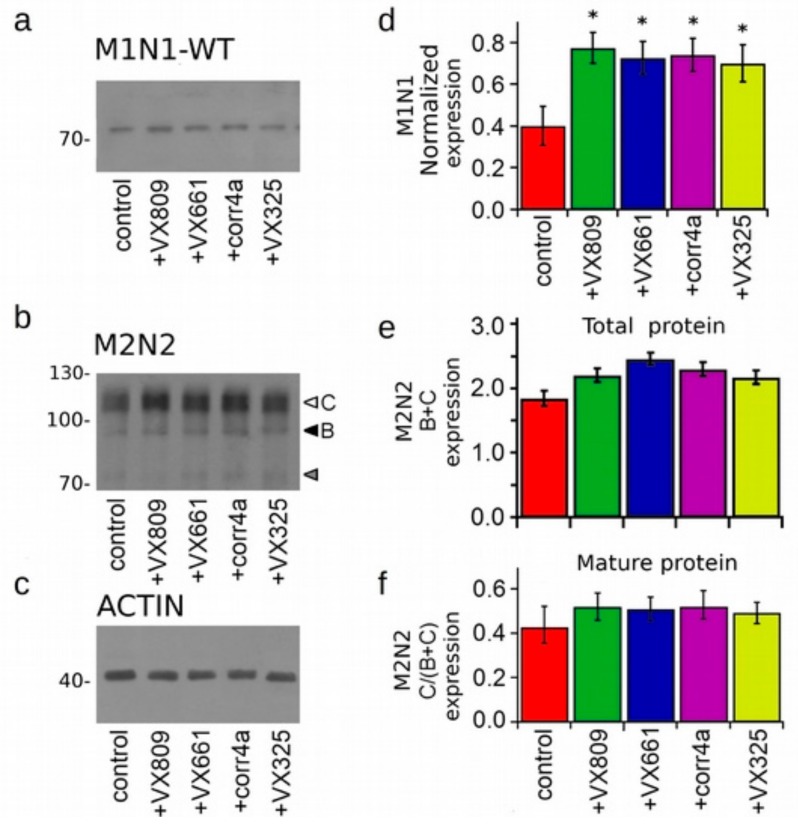
Expression of the WT-CFTR N-half co-expressed with the C-half. (**a**) Detection of WT-M1N1 in whole cell extracts of HEK-t cells co-transfected with WT-M1N1 + M2N2 and treated with DMSO (control) or with 5 µM VX809, 5 µM VX661, 10 µM corr4a and 5 µM VX325. (**b**) The M3A7 antibody, raised against the NBD2 domain, recognized the M2N2 polypeptide in the same whole cell lysates. (**c**) Expression level of the housekeeping protein actin in the same samples in (**a**) and in (**b**). The molecular weights of the proteins of the molecular weight marker that was run in the SDS-PAGE are shown on the left of each blot. (**d**) Normalized expression of the WT-M1N1 polypeptide. (**e**) Bar graph indicating the quantification of the total M2N2 protein expression, calculated as the sum of band B and C, normalized by the actin expression. (**f**) Bar graph indicating the quantification of the mature, fully glycosylated fraction of the M2N2 protein, expressed as the C/(C + B) band ratio. Data are expressed as mean ± standard error of the mean (SEM) of at least four independent experiments. Asterisks indicate a statistical significance versus the control: * *p* < 0.05.

**Figure 5:**
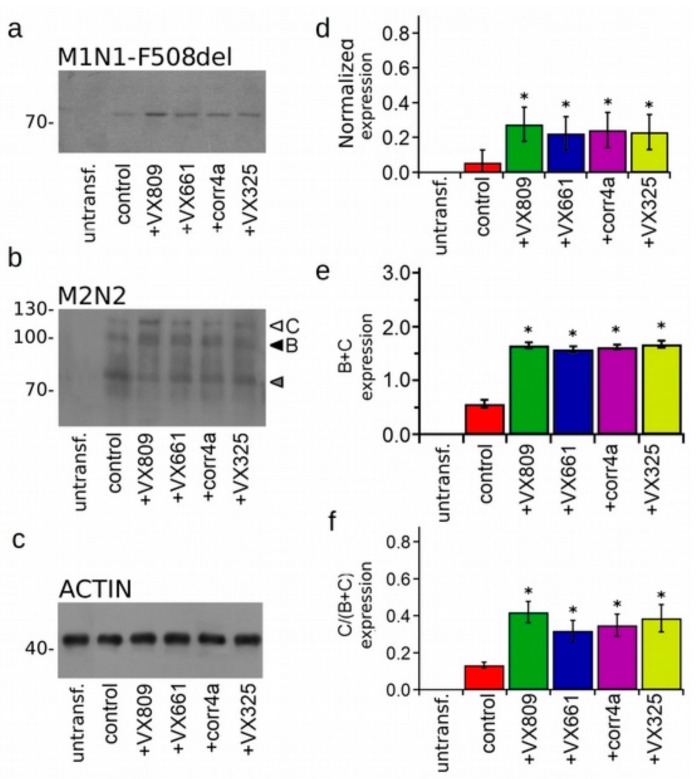
Expression of the F508del-CFTR N-half co-expressed with the C-half. (A) Detection of M1N1-F508del (**a**) and M2N2 (**b**) in whole cell lysates of untransfected (untransf.) and transiently co-transfected HEK-t cells treated with DMSO (control), VX809, VX661, corr4a and VX325. (**c**) Expression of the protein actin in the same samples in (**a**) and in (**b**). The molecular weights of the proteins of the molecular weight marker that was run in the SDS-PAGE are shown on the left of each blot. (**d**) Normalized expression of the F508del-M1N1 polypeptide. (**e**) Bar graph indicating the quantification of the total M2N2 protein expression, calculated as the sum of bands B and C, normalized by the actin expression. (**f**) Bar graph indicating the quantification of the mature, fully glycosylated fraction of the M2N2 protein, expressed as the C/(C + B) band ratio. Data are expressed as mean ± standard error of the mean (SEM) of at least four independent experiments. Asterisks indicate a statistical significance versus the control: * *p* < 0.05.

To further test the effect of correctors on the expression of the CFTR halves, M1N1 (WT or F508del) and M2N2 polypeptides were co-transfected in HEK-t cells, and, after treatment with correctors, whole cell extracts were subjected to an SDS-PAGE immunoblot analysis. Figure 4a and Figure 5a show that the monoclonal antibody MM13-4 raised against the N-terminal of the CFTR polypeptide was able to detect WT- and F508del-M1N1 as a band of ~72 KDa, corresponding to the predicted size of this construct. Analogously to what was observed when the M1N1 polypeptide was transfected alone, the VX809, VX661 and VX325 correctors determined a significant increase of the expression either of WT- and F508del-M1N1 molecules (Figure 4d and Figure 5d). Intriguingly, in these preparations, the corr4a corrector was also able to significantly enhance the expression of both WT- and F508del-M1N1.

Same whole cell extracts from M1N1 + M2N2 co-transfected HEK-t cells were subsequently used to perform an immunoblot analysis aimed to evaluate the expression of the M2N2 protein. As the CFTR C-half includes the two glycosylation sites of the CFTR protein, this polypeptide was detected by the M3A7 antibody raised against the NBD2 domain as two electrophoretic bands (white and black head arrows in Figure 4b and Figure 5b). For both the WT- and F508del- isoforms, the higher molecular weight band (C band) of ~110 KDa, corresponded to the mature, fully glycosylated form of the M2N2 polypeptide, while the lower molecular weight (B band) of ~90 KDa, was the core-glycosylated or partially glycosylated form. In both preparations, a supplementary band of ~73 KDa (gray arrow in Figure 4b and Figure 5b), probably corresponding to the unglycosylated C-half, was also detected. This band was not considered in the quantification analysis. In the WT-M1N1 + M2N2 whole cell lysates, the corrector treatment did not enhance either the total expression or the maturation of the fully glycosylated mature form of the M2N2 polypeptide, as displayed by the bar graphs in Figure 4e,f. On the contrary, in the F508del-M1N1 + M2N2 whole cell lysates, correctors demonstrated the ability to increase both the total expression (B + C band) and the maturation (C/(C + B) band ratio) of the M2N2 polypeptide (Figure 5e,f).

**Figure 6:**
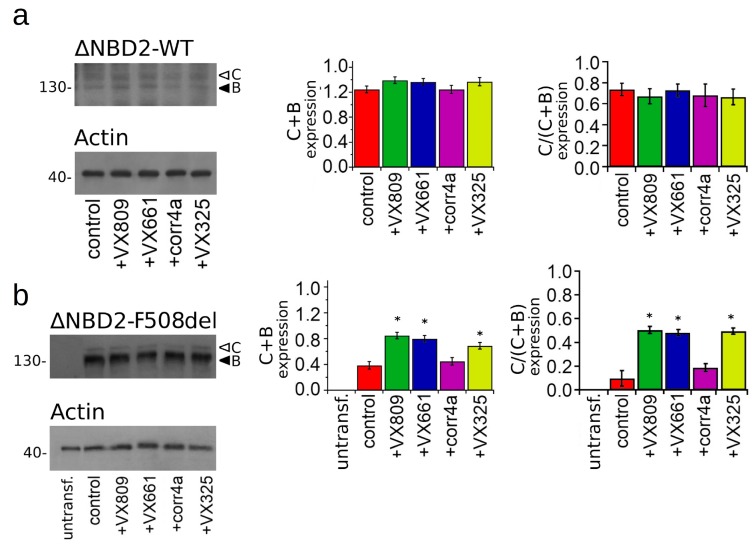
Biochemical analysis of WT- and F508del-ΔNBD2 expression pattern. (**a**) Electrophoretic mobility of WT-ΔNBD2 in untreated HEK-t cells (control) or treated for 18 h with the VX809, VX661, corr4a and VX325 correctors. (**b**) Expression of F508del-ΔNBD2 in untransfected (untransf.) and transiently transfected cells after 18 h of treatment with DMSO (control) or with 5 µM VX809, 5 µM VX661, 10 µM corr4a and 5 µM VX325. Arrowheads indicate the fully-glycosylated (C band), the core-glycosylated (B band), and the unglycosylated, immature forms of the ΔNBD2 proteins. In the lower blots, the expression of actin, used as housekeeping protein, is shown. The molecular weight of the proteins of molecular weight marker that was run in the SDS-PAGE is indicated on the left of each blot. Bar graphs in the middle show the quantification of the total ΔNBD2 protein expression, calculated as the sum of the B and C bands, normalized by the actin expression. Bar graphs on the right indicate the quantification of the mature, fully glycosylated fraction of the ΔNBD2 protein, expressed as the C/(C + B) band ratio. Data are expressed as mean ± SEM of at least four independent experiments. Statistical significance was tested by Dunnet multiple comparisons test (all groups against the control group). Asterisks indicate statistical significance versus DMSO: * *p* < 0.05.

Finally, we tested the effect of the correctors on the expression of the WT- and F508del CFTR that lacked NBD2. Regarding the glycosylation sites of CFTR in this construct, in a way that was analogous to the full length CFTR, the ∆NBD2 was detected in whole cell lysates of the HEK-t transfected cells as two electrophoretic bands of ~130 KDa (B band, immature, partially glycosylated) and ~150 KDa (C band, mature, fully glycosylated) (Figure 6). The bar graph of Figure 6a shows that none of the tested correctors were able to enhance the WT-∆NBD2 total expression (B + C band) or increase the expression of its mature, fully glycosylated form (C/(C + B) band ratio). On the contrary, in lysates from the F508del-∆NBD2 HEK-t transfected cells, the treatment with the VX809, VX661 and VX325 correctors determined a significant increase of either total protein (C + B band) or mature protein fraction (C/(C + B) band ratio) expression with respect to the control, untreated cells. In F508del-∆NBD2 preparations, the corr4a corrector did not elicit an augmentation of either of the total expression (C + B band) or of the maturation grade of the protein (C/(C + B) band ratio).

### 2.4. Effect of Correctors on the Stabilization of CFTR N-Half.

**Figure 7:**
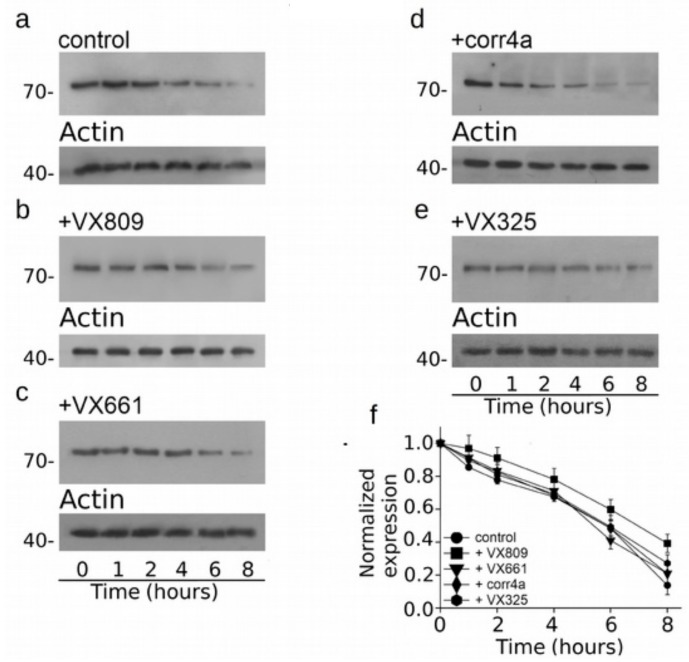
Evaluation of WT-M1N1 stability by means of the cycloheximide chase approach. Expression of the WT-M1N1 polypeptide in HEK-t cell lysates treated with DMSO (**a**), 5 µM VX809 (**b**), 5 µM VX661 (**c**), 10 µM corr4a (**d**) and 5 µM VX325 (**e**) and subjected to protein synthesis inhibition by means of incubation with 0.5 mg/mL of cycloheximide. The lanes of each blot represent six different time points as indicated at the bottom of the figure. For each condition, the expression of the protein actin is shown in the lower panels of (**a**–**e**). (**f**) Expression of the WT-M1N1 protein at each time point. Data are expressed as mean ± SEM of at least four independent experiments. In the legend of the figure are indicated the symbols used to represent each corrector used in the experiment. For all conditions under analysis, the amount of the WT-M1N1 protein was normalized to actin and expressed relative to time 0.

**Figure 8:**
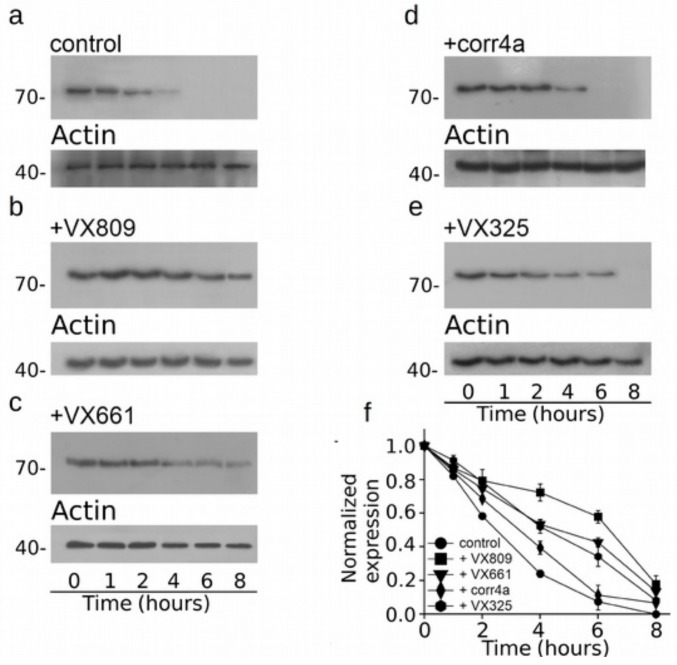
Cycloheximide chase experiments to assess the stability of the M1N1-F508del protein. Western blot images revealing the stability of M1N1-F508del in whole cell lysates from transfected HEK-t cells treated with DMSO, 5 µM VX809, 5 µM VX661, 10 µM corr4a and 5 µM VX325 at various time intervals after incubation with cycloheximide are shown in (**a**–**e**), respectively. At the same time points, the expression of the protein actin is shown at the bottom of each panel. (**f**) Evaluation of the relative expression of the M1N1-F508del protein after 0, 1, 2, 4, 6 and 8 h from the blockade of protein synthesis by incubation with cycloheximide. In each condition, the M1N1-F508del expression was normalized to that of actin and referred as relative to time 0. Data are expressed as mean ± SEM of at least four independent experiments.

As three out of four correctors that we tested seem to mostly affect the expression of the CFTR N-half, we assessed whether the action of correctors on this region could be also linked to the augmentation of its stability. Figure 7 presents the cycloheximide chase experiments, showing that the expression of WT-M1N1 decays to 48% after 6 h and to 13% after 8 h from the beginning of the treatment with cycloheximide. Interestingly, after 6 and 8 h from the blockade of protein synthesis with cycloheximide, the expression level of WT-M1N1 treated with VX809 was in 60% and 40% of the initial one, respectively. The other analyzed correctors did not exert any effect on the stability of the CFTR N-half (Figure 7b–f). In Figure 8a, it is shown that F508del-M1N1 was less stable than WT-M1N1 over time. In fact, the expression level of the control, untreated polypeptide decayed to 24% of its initial level 4 h after the administration of cycloheximide and to an almost negligible level after 6 h. All correctors, except corr4a, determined a significant increase of the stability of F508del-M1N1 (Figure 8b–f). The most efficient corrector was VX809. In fact, the expression level of the CFTR N-half was the 72% and 58% of the initial one after 4 and 6 h from the beginning of the treatment with cycloheximide, respectively (Figure 8f). The expression level of F508del-M1N1 incubated with VX661 and VX325 was the 53% and 52% after 4 h and the 43% and 34% after 6 h, respectively. Tables S6 and S7 report, for each time interval depicted in Figure 7 and Figure 8, the WT- and F508del-M1N1 protein expression data normalized for the housekeeper protein actin and their statistical significance, as tested by Dunnet multiple comparisons test (all groups against the control group).

## 3. Discussion

Cystic fibrosis is caused by genetic mutations that lead to the reduced activity or expression of the CFTR at the cell surface of epithelial cells. The majority of CF patients express at least one allele that bears the F508del mutation, which causes a processing defect that impairs folding, trafficking, stability and CFTR function as a chloride and bicarbonate ion channel [50].

Since several in vivo and in vitro studies have suggested that enhancing the F508del-CFTR functional activity as little as 5% may significantly diminish CF symptoms [51,52,53], a major effort of CF research has been intended to identify and characterize small molecules, namely correctors, that are able to deliver defective processing CFTR mutants (class 2 mutations), such as the F508del-CFTR, at the plasma membrane. The rationale has been that once rescued for their folding and trafficking defects, these proteins, opportunely stimulated by a potentiator, would result in functional channels. However, the correctors identified to date, such as lumacaftor (VX809) [23], VX325 [54] or corr4a [18] have turned out to elicit still too low therapeutic effect [55,56]. The knowledge of the mechanism of action of currently available correctors would undoubtedly aid in the development of more effective molecules.

In the present study, we focused on the mechanisms by which four different correctors, VX809, corr4a, VX325 and the new approved derivative VX661, act to increase the expression of the mutant CFTR on the plasma membrane. In this study, we exploited the properties of the heterologous expression systems (HEK-t and FRT cells) to obtain a high expression rate of the transfected constructs, and this was compared with the enhanced response of the transfected CFTR channel. We asked the following questions: (1) Do tested correctors act in the same way on the mutant F508del- and WT-CFTR proteins? (2) Do the protein expression levels of the corrector-rescued F508del- and WT-CFTR correlate with their chloride channel activity? (3) Which region of the CFTR protein is mainly affected by the action of correctors? (4) Do the tested correctors use the same mechanism to increase the expression of the CFTR molecule? Before addressing these questions, we ascertained whether the VX809, VX661, corr4a and VX325 correctors could influence the expression of the RNAs that codified for the constructs that we used in this study. To achieve this aim, the expression level of the transcripts of the WT- and F508del-CFTR, WT- and F508del-∆NBD2, the CFTR C-half and the WT- and F508del-CFTR N-halves transfected in HEK-t cells alone or co-expressed with the CFTR C-half were evaluated in each preparation by real-time PCR (see Tables S1–S5). The RNA coding for all the constructs that we used was approximately constant in all preparations, independently from the treatment (or not) with correctors; therefore, we could conclude that correctors exert their effect on the expression of the CFTR constructs at the post-transcriptional level.

Though the capability of VX809, corr4a and VX325 to enhance the surface expression of the mutant F508del-CFTR has been largely tested, to our knowledge, no comparative analysis of the effects of these molecules either on WT and mutant isoforms of the CFTR has been accomplished. Furthermore, no data regarding the mechanism of action of the new approved molecule VX661 have yet been provided. To test whether correctors exert the same effect on the expression level of the WT and mutant F508del-CFTR, we compared the expression level of total protein (B + C band) and the amount of the mature form (C/(C + B) band ratio) of the SDS-PAGE whole cell lysates obtained from the control and corrector-treated samples. Our analysis showed that in WT-CFTR lysates, the two considered parameters did not increase after treatment with correctors (Figure 1A). On the contrary, both total protein and mature protein expression levels of the F508del-CFTR were significantly enhanced after incubation with each of the four correctors under analysis. Intriguingly, the difference of the effect of correctors on the expression levels of the WT- and F508del-CFTR proteins let us hypothesize that correctors may directly act on those regions of the F508del-CFTR whose folding is different from the WT [34,38] or, alternatively, may modulate any component of the cellular machinery responsible for defective protein degradation, such as the ERAD, preventing the mutant CFTR from premature disruption or even inducing the ER quality control complex to recognize it as a protein that is prone to leave the ER compartment. Other interesting findings of the present work concern the analysis of the capability of the WT and F508del-CFTR isoforms incubated with correctors to transport iodide. Similarly to what emerged from our analysis of protein expression, the forskolin-stimulated WT-CFTR iodide transport activity was not modified by treatment with correctors (Figure 2A). Taken together with the results regarding the protein expression level, these data suggest that in WT-CFTR cells, either the expression and the channel activity of the CFTR protein reached a steady-state level that could not be further incremented by means of a drug-mediated approach. The iodide transport of the F508del-CFTR-transfected FTR cells was very reduced and even if the mutant channel activity was significantly increased after treatment with correctors, it did not reach the level of the WT-CFTR (Figure 2B). The channel transport activity of the corrector-treated F508del-CFTR FRT cell preparations followed the order of VX809 > VX661 > VX325 ≥ corr4a. Except for VX661, whose activity has not been previously tested, these results are in agreement with chamber recordings of transepithelial currents elicited in HBE-F508del cells [23]. The obtained results indicate that the amelioration of the processing defects exerted by correctors positively correlates with the increased activity of the F508del-CFTR as a channel. The fact that, even after treatment with correctors, the expression of the F508del-CFTR did not reach the level of WT once more shows the impelling necessity to develop cystic fibrosis therapeutics with a higher target affinity as well as an improved corrective efficacy.

Another question that we addressed in this study regarded the identification of the CFTR regions that are mainly affected by the action of correctors. NBDs have been indicated as possible correctors’ target sites. Some studies have suggested that VX809 and VX325 directly act on NBD1 [44,57], while other research groups have provided evidence that these two correctors restore the F508del-CFTR defective NBD1:ICL4 interactions [32,33,39,40]. Some mutagenesis experiments have evidenced that the presence of NBD2 is necessary for corr4a to exert its maximal effect [42]. Another possibility is that correctors promote the folding of TMDs. VX809 has been found to be able to bind and stabilize TMD1 [34], to promote the interaction between NBD1 and ICL1 [36], and to favor interactions among TMD1, TMD2 and NBD1 [38]. Finally, the hypothesis that correctors do not directly interact with the CFTR protein but affect the CFTR cellular quality control mechanisms cannot be excluded.

Aware that the co-expression of the two CFTR hemi-molecules yields functional CFTR chloride channels at cell surfaces [58,59,60], to recognize the regions whose expression is mainly modulated by correctors, we generated expression constructs containing CFTR N-half (residues 1–533), either in its WT and F508del isoforms, and C-half (residues 837–1480). The regulatory domain was not comprised, as it has been demonstrated that its deletion does not affect the maturation and trafficking to the cell surface of CFTR mutants which lack this domain [61,62].

In agreement with previous reports by Clarke’s group [33,34], we found that the C-half protein did not express the C band corresponding to the mature fully glycosylated form of the protein, unless it was co-expressed with the N-half (see Figure S1). When it was co-expressed with its counterpart, M2N2 showed either B and C bands, whose expression was almost equal in all WT-M1N1 + M2N2 preparations. On the contrary, correctors were able to increase the total expression level as well as the expression level of the C band of F508del-M1N1 + M2N2 whole cell lysates (Figure 4 and Figure 5). These results confirm Clarke’s group findings, which showed that the corr4a and VX325 correctors enhance the expression and maturation of F508del-M1N1 + M2N2 co-expressed constructs [33] and further demonstrate that the VX809 and VX661 correctors are able to exert the same effect on the CFTR hemi-halves.

We then evaluated the effect of correctors on the expression level of the CFTR N-half. This construct was successfully used to demonstrate that the VX809 corrector both acts by stabilizing TMD1 [34] and by correcting F508del-CFTR folding defects through its action on TMD1 [38]. Our results indicate that this is the region mostly affected by the action of the analyzed correctors. In fact, VX809, VX661 and VX325 significantly increased the expression of both WT- and F508del-M1N1 isoforms both when they were expressed alone and together with the C-half. By contrast, corr4a was able to enhance the expression of the M1N1 polypeptide only when it was co-expressed with M2N2. These results indicate that the second half of the CFTR is necessary for corr4a to exert its effect on the expression of the CFTR molecule. To definitively confirm that within the CFTR C-half, the NBD2 domain is effectively the target of corr4a, as pointed out by the works of Cyr’s group [42], we compared the expression level of a CFTR construct that lacked this domain but still maintained the capability to express both CFTR bands corresponding to the mature, fully glycosylated (B band) and to the immature, the core glycosylated forms of the CFTR protein (C band) [63] before and after treatment with correctors. The construct lacking NBD2 was successfully used by Christine Bear’s group to test the mechanism of the action of trimethylangelicin, which was demonstrated to be both a potentiator of the WT-CFTR [64] and a corrector of the F508del-CFTR [65]. Figure 6a shows that both the total protein and mature protein expression levels of WT-∆NBD2 were not modified by the treatment with correctors, while the folding and trafficking defects of the F508del isoform were rescued by the treatment with the VX809, VX661 and VX325 correctors, resulting in a significant increase of the level of the total protein (B + C band) and mature protein fraction ((C/C + B) band ratio) of F508del-∆NBD2 in comparison to the control, untreated sample. By contrast, corr4a did not enhance the total expression and did not promote the maturation of the F508del-∆NBD2 protein (Figure 6b). Our findings corroborate previous literature’s evidence that NBD2 is not necessary for the action of the VX809, VX661 and VX325 correctors. On the contrary, this domain was revealed to be the target region of the bithiazole compound corr4a. It has also to be noticed that, similarly to what we postulated for the full length WT- and F508del-CFTR, correctors seem to be able to discriminate between the WT- and F508del-∆NBD2 isoforms, specifically recognizing the defective conformation of the region in which F508del is inserted.

The last task that we tried to face in this work was in regard to the disclosure of the mechanisms through which correctors increase the expression of the CFTR protein. As the CFTR N-half was the region mostly affected by the action of the correctors under study, we decided to focus on this region to start to answer this question. We hypothesized that if correctors act by stabilizing the region whose expression they increase, then it would be expected that the M1N1 region would have a slower turnover rate after transiently transfected cells were treated with cycloheximide to inhibit protein synthesis. HEK-t cells were transfected with WT- or F508del-M1N1 and incubated in the presence of VX809, VX661, corr 4a, VX325, or DMSO. The next day, cycloheximide was added to inhibit protein synthesis. Whole cell extracts were collected at various time points and subjected to immunoblot analysis. As expected, in the absence of correctors, the stability of F508del-M1N1 was reduced with respect of that of WT (Figure 7 and Figure 8, Tables S6 and S7). The most effective among the CFTR corrector drugs under analysis was VX809, which stabilized the CFTR N-half independently from the isoform (WT- or F508del-) that was expressed in cycloheximide treated cells (Figure 7 and Figure 8, Tables S6 and S7). The other three correctors only enhanced the turnover rate of the F508del-M1N1 polypeptide, prompting us to argue that their action on the expression of the WT-M1N1 isoform passes through mechanisms that fall outside the stabilization of the protein half-life. An important unsolved question that our stability experiments raise is in regard to the compartment involved in the change of M1N1 stability. Since this CFTR segment was not glycosylated, it is necessary to set up more refined biochemical and molecular biology experiments that are currently under design and study in order to determine if the change happens at the ER or in the cell periphery.

In summary, three major conclusions can be derived from the results of this study. First, correctors show a different behavior towards full length WT- and F508del-CFTR molecules, determining an increase only of the functional expression of the mutant isoform. Second, although we have not provided any direct evidence that correctors directly bind to the F508del-CFTR, we have provided evidence that correctors selectively affect the expression of determined CFTR regions: The VX809, VX661 and VX325 correctors increase the expression of the F508del-CFTR N-half, while the corr4a corrector positively influences the expression of the C-half of the molecule and of the NBD2 domain in particular. Third, the CFTR N-half was revealed to be a hotspot region whose expression and stabilization properties should be kept into deep consideration in the search for more effective corrector therapeutics.

## 4. Materials and Methods

### 4.1. Chemicals

The 3-[6-[[[1-(2,2-difluoro-1,3-benzodioxol-5-yl)cyclopropyl]carbonyl]amino]-3-methyl-2-pyridinyl]-benzoic acid (Lumacaftor, VX809), 1-(2,2-difluoro-1,3-benzodioxol-5-yl)-N-[1-[(2R)-2,3-dihydroxypropyl]-6-fluoro-2-(2-hydroxy-1,1-dimethylethyl)-1H-indol-5-yl]-cyclopropanecarboxamide (Tezacaftor, VX661), N-[2-(5-chloro-2-methoxyphenylamino)-2 0-yl]benzamide (corr4a), 4-(cyclohexyloxy)-2-{1-[4-(4-metho-xybenzenesulfonyl)piperazin-1-yl]ethyl}quinazoline (VX325) correctors were purchased from Selleck Chemicals (Munich, Germany). If not explicitly indicated in the text, all other chemicals and culture media components were provided by Sigma-Aldrich (Milan, Italy).

### 4.2. Cell Culture

Human, highly transfectable embryonic kidney 293 (HEK-t) cells were purchased from the Interlab Cell Line Collection (Genoa, Italy). Cells were grown in Dulbecco’s modified Eagle’s medium (DMEM) supplemented with 2 mM l-glutamine, 1% PenStrep (100 U/mL) and 20% FBS at 37 °C and 5% CO_2_. To prevent the loss of differentiation potential, cells were not allowed to become confluent. Fisher rat thyroid (FRT) cells were stably co-transfected with a halide-sensitive yellow fluorescent protein (YFP-H148Q/I152L; [66,67]), and the WT- or F508del-CFTR were cultured in Coon’s modified medium supplemented with 10% FBS, 2 mM l-Glutamine, 1% PenStrep (100 U/mL), 1 mg/mL geneticin (G418), and 0.6 mg/mL zeocin as selection agents at 37 °C and 5% CO_2_.

### 4.3. Generation and Expression of CFTR Constructs

Plasmids containing the CFTR N-half (M1N1, residues 1–633) or the C-half (M2N2, residues 837–1480) molecules, as well as the constructs codifying for the whole CFTR (residues 1–1480) and the CFTR lacking the NBD2 domain (∆NBD2, residues 1–1172) were sub-cloned between the Hind III and XhoI restriction sites of the expression vector pCDNA3 (Invitrogen, Paisley, UK), respectively. The cDNAs encompassing the phenylalanine at position 508 of the CFTR molecule were further modified by site-directed mutagenesis to introduce the F508del deletion, using the QuickChange kit (Stratagene, Santa Clara, CA, USA). The mutation was verified by sequencing (Biofab Research, Rome, Italy)

For transfection, HEK293-t cells were plated onto poly-l-lysine-coated culture dishes and grown to 65% confluence in a complete medium. Cells were transiently transfected using Lipofectamine 2000 (Invitrogen, Paisley, UK) with 4 μg of cDNA coding for the full length CFTR, M1N1, M2N2 and ∆NBD2 or with 2 μg of the cDNAs coding for M1N1 plus 2 μg of the construct encoding M2N2. The transfection medium (DMEM supplemented with 2 mM l-glutamine) was replaced after 6 h with a fresh complete medium containing 5 µM VX809, 5 µM VX661, 10 µM corr4a, 5 µM VX325 or vehicle DMSO (control). Cells were harvested after 24 h.

### 4.4. RNA Isolation, Reverse Transcription, and Quantitative Real-Time Polymerase Chain Reaction

Total RNA was isolated using the RNeasy Mini kit (Qiagen, Hilden, Germany), and first-strand cDNA was synthesized from 2 µg of RNA using the RevertAid First Strand cDNA Synthesis Kit and random hexamers according to the manufacturer’s instructions (Fermentas, Burlington, ON, Canada). First-strand cDNA from transfected HEK293-t cells was employed as the template in a quantitative real-time polymerase chain reaction (qRT-PCR) in a CFX Connect Real-Time PCR Detection System instrument (Bio-Rad Laboratories, Hercules, CA, USA). The sequences of the oligonucleotide primer pairs specific for the full length CFTR, M1N1, M2N2, and ∆NBD2 and the amplification conditions are listed in Table S8. Changes in cDNA amount were evaluated using the comparative cycle threshold (*C*t) method. Each sample was run in at least quadruplicate.

### 4.5. Western Blot

Cells were lysed in a RIPA lysis buffer (50 mM Tris-HCl, pH 8.0, 150 mM NaCl, 1% Triton X-100, 1% sodium deoxycholate, 0.1% SDS) containing a complete protease inhibitor cocktail (Sigma-Aldrich). Protein concentration was determined by Bradford’s method by using bovine serum albumin as the standard. Equal amounts of proteins (30 μg) were subjected to SDS 10% polyacrylamide gel electrophoresis and transferred to a PVDF membrane (Millipore, Billerica, MA, USA) for 1 h. Blots were incubated with an anti-CFTR monoclonal primary antibody raised against residues 25–36 in the N-terminal of the CFTR protein (clone MM13-4, Millipore, dilution 1:1000;) to detect the full length CFTR, M1N1 and ∆NBD2; with an anti-CFTR monoclonal primary antibody raised against the C-terminal of the NBD2 domain (clone M3A7, Millipore, dilution 1:1000) to detect M2N2; and with horseradish peroxidase conjugated the goat anti-mouse antibody (dilution 1:2000; Santa Cruz Biotechnologies, Dallas, TX, USA) as a secondary antibody. Immunodetection was performed using Amersham ECL PLUS detection reagents (GE Healthcare Europe, Milan, Italy), and the images were captured by using Amersham Hyperfilm ECL. In order to confirm the homogeneity of the loaded proteins, immunoblots were stripped by incubating them with stripping buffer (62.5 mM Tris-HCl, pH 6.8, 10% SDS and 1% β-mercaptoethanol) for 30 min at 55 °C and re-probed with an anti-actin polyclonal antibody (1:2000, Sigma). Untransfected cells lysate was assayed with both anti-CFTR antibodies as a negative control. For quantification, western blot images were analyzed with the software ImageJ (U.S. National Institutes of Health, Bethesda, MD, USA). For each lane, the bands, analyzed as regions of interest, were quantified and normalized to the intensity of the band corresponding to the actin detected in the stripped PVDF membranes. The western blotting of each analyzed condition was repeated at least in 4 independent experiments. Controls in untransfected HEK-t cells showed that none of the used antibodies detected the presence of CFTR polypeptides in the blots (data not shown).

### 4.6. Iodide Influx Assay

Fischer rat thyroid (FRT) cells were stably co-transfected with a halide-sensitive yellow fluorescent protein (YFP-H148Q/I152L), and the WT- and F508del-CFTR were cultured in standard conditions (37 °C, 5% CO_2_) on black-wall, clear bottom 96 well micro-plates at a density of 30,000 cells per well. The day after seeding, cells were incubated for 16–18 h with 5 µM VX809, 5 µM VX661, 10 µM corr4a, 5 µM VX325, or DMSO as a control. The assay was based on the fact that the YFP protein fluorescence is quenched to a greater extend by I^−^ than by Cl^−^ [66]. The influx of iodide mediated by the transport activity of the forskolin-activated CFTR was measured as a quenching of the YFP protein [68] using a fluorescence plate reader (Tristar2 S, Berthold Technologies, Bad Wildbad, Germany) equipped with 485 nm excitation and 535 nm emission filters. If not otherwise stated, 40 min before the assay, the cells were washed twice with a solution containing (in mM): NaCl 136, KNO_3_ 4.5, Ca(NO_3_)_2_ 1.2, MgSO_4_ 0.2, Glucose 5, HEPES 20, (pH 7.4). The cells were then incubated in 60 μL of this solution at 37 °C for 25 min with (or without) 20 µM forskolin to activate the CFTR protein.

Once the assay started, the fluorescence was recorded every 0.2 s for as long as 25 s for each well. Two seconds after the beginning of the recording of the fluorescence, 100 μL of an extracellular solution containing 136 mM NaI instead of NaCl was injected so that the final concentration of NaI in the wells was 85 mM. The iodide influx was detected as a fluorescence quenching as the I^−^ anion binds to the intracellular YFP. The initial rate of fluorescence decay (QR) was derived by fitting the signal with an exponential function after background subtraction and normalization for the average fluorescence before NaI addition.

### 4.7. Cycloheximide Chase Assay

To evaluate the stability of the WT- and F508del-M1N1 proteins, the HEK293-t cells were transfected with the plasmids containing the cDNAs encoding the WT- or F508del-CFTR N-half (residues 1–533) and incubated for 18 h in the presence of DMSO (control), 5 μM VX809, 5 μM VX661, 10 μM corr4a, or 5 μM VX325. Protein synthesis was then inhibited by addition of 0.5 mg/mL cycloheximide. At 6 different time points (after 0, 1, 2, 4, 6, and 8 h) the cells were harvested, and samples of whole cell SDS extracts were subjected to immunoblot analysis with monoclonal antibody MM13-4 raised against the N-terminus of the CFTR protein.

### 4.8. Statistics

Data were analyzed using the Igor software (version 8.0.3.3, Wavemetrics, Lake Oswego, OR, USA). Results are expressed as mean ± SEM (standard error of the mean). A multiple comparison Dunnet test was used to compare data sets. In all cases, significance was accepted for a probability of *p* < 0.05.

## Supplementary Materials

The following are available online at https://www.mdpi.com/1422-0067/20/21/5463/s1.
