# Supplementary material for: Unravelling the Regions of Mutant F508del-CFTR More Susceptible to the Action of Four Cystic Fibrosis Correctors
Source: Int J Mol Sci. 2019 Nov 1;20(21):5463. doi: 10.3390/ijms20215463 (PMC6862496; doi:10.3390/ijms20215463)
Supplement: Supplementary file 1 [file ijms-20-05463-s001.pdf]

Unravelling the regions of mutant F508del-CFTR more susceptible to the action of four cystic fibrosis correctors

Giulia Amico, Chiara Brandas, Oscar Moran and Debora Baroni

Istituto di Biofisica, CNR. Via De Marini, 6. 16149 Genova, Italy

Supplementary materials

Table S1. Evaluation of CFTR mRNA abundance in HEK-t cells transfected with WT or F508del CFTR after treatment with VX809, VX661, corr4a and VX325 correctors. The mRNA abundance is expressed as the cycle threshold (Ct) obtained by real-time PCR and normalized to the expression of the GAPDH used as housekeeping gene. Data represent the mean± sem (standard error of the mean). For each condition, the number of observations is indicated into the brackets. Comparisons of values with those of control (untreated) cells were made with the Dunnet test. Probability p values are reported. Differences are significant when $p < 0.05$.

	**WT-CFTR**		**F508del-CFTR**	
	**normalised Ct**	**p**	**normalised Ct**	**p**
Control	0.82 ± 0.14 (4)		0.95 ± 0.31 (5)	
+ VX809	0.93 ± 0.20 (4)	>0.99	0.95± 0.30 (6)	>0.99
+ VX661	0.93 ± 0.19 (4)	0.92	0.94 ± 0.20 (5)	>0.99
+ corr4a	0.87 ± 0.05 (4)	0.68	0.81 ± 0.31 (4)	>0.99
+ VX325	0.94 ± 0.06 (4)	0.63	0.76 ± 0.37 (4)	0.91

Table S2. Evaluation of M1N1 mRNA abundance in HEK-t cells transfected with WT or F508del M1N1 after treatment with VX809, VX661, corr4a and VX325 correctors. The mRNA abundance is expressed as the cycle threshold (Ct) obtained by real-time PCR and normalized to the expression of the GAPDH used as housekeeping gene. Data represent the mean ± sem (standard error of the mean). For each condition, the number of observations is indicated into the brackets. Comparisons of values with those of control (untreated) cells were made with the Dunnet test. Probability p values are reported. Differences are significant when $p < 0.05$.

	**WT-M1N1**		**F508del-M1N1**	
	normalised Ct	p	normalised Ct	p
Control	0.98 ± 0.20 (4)		1.03 ± 0.13 (6)	
+ VX809	0.99 ± 0.24 (4)	>0.99	0.98 ± 0.07 (4)	0.55
+ VX661	1.07 ± 0.20 (4)	>0.99	1.16 ± 0.13 (6)	0.94
+ corr4a	1.11 ± 0.29 (4)	>0.99	0.99 ± 0.25 (5)	0.99
+ VX325	0.86 ± 0.23 (5)	0.99	0.92 ± 0.14 (6)	0.97

Table S3. Evaluation of M2N2 mRNA abundance in HEK-t cells transfected with M2N2 after treatment with VX809, VX661, corr4a and VX325. The mRNA abundance is expressed as the cycle threshold (Ct) obtained by real-time PCR and normalized to the expression of GAPDH used as housekeeping gene. Data represent the mean ± sem (standard error of the mean). For each condition, the number of observations is indicated into the brackets. Comparisons of values with those of control (untreated) cells were made with the Dunnet test. Probability p values are reported. Differences are significant when $p < 0.05$.

	**M2N2**	
	normalized Ct	p
Control	0.58 ± 0.04 (4)	
+VX809	0.58 ± 0.06 (4)	0.73
+VX661	0.52 ± 0.07 (4)	>0.99
+corr4a	0.50 ± 0.08 (4)	>0.99
+VX325	0.59 ± 0.05 (4)	0.96

Table S4. Evaluation of M1N1 and M2N2 mRNA abundance in HEK-t cells transfected with WT or F508del M1N1 and M2N2 after treatment with VX809, VX661, corr4a and VX325 correctors. The mRNA abundance is expressed as the cycle threshold (Ct) obtained by real-time PCR and normalized to the expression of the GAPDH used as housekeeping gene. Data represent the mean ± sem (standard error of the mean). For each condition, the number of observations is indicated into the brackets. Comparisons of values with those of control (untreated) cells were made with the Dunnet test. Probability p values are reported. Differences are significant when $p < 0.05$.

	**WT-M1N1**		**M2N2**	
	**normalised Ct**	**p**	**normalised Ct**	**p**
Control	1.09 ± 0.07 (5)		1.10 ± 0.09 (5)	
+ VX809	1.01 ± 0.10 (4)	0.75	1.15 ± 0.10 (4)	0.89
+ VX661	1.00 ± 0.17 (5)	0.94	1.15 ± 0.22 (4)	0.77
+ corr4a	1.04 ± 0.21 (5)	0.98	1.20 ± 0.20 (4)	0.30
+ VX325	0.94 ± 0.16 (6)	>0.99	1.12 ± 0.16(4)	0.25

	**F508del-M1N1**		**M2N2**	
	**normalised Ct**	**p**	**normalised Ct**	**p**
Control	0.99 ± 0.08 (5)		1.22 ± 0.06 (4)	
+ VX809	1.01 ± 0.09 (4)	0.27	1.21 ± 0.08 (4)	0.89
+ VX661	0.93 ± 0.09 (4)	0.45	1.21 ± 0.07 (5)	0.95
+ corr4a	0.92 ± 0.08 (4)	0.98	1.40 ± 0.09 (6)	0.74
+ VX325	1.02 ± 0.04 (4)	0.39	1.33 ± 0.13 (4)	0.70

Table S5. Evaluation of ΔNBD2 mRNA abundance in HEK-t cells transfected with WT or F508del ΔNBD2 after treatment with VX809, VX661, corr4a and VX325 correctors. The mRNA abundance is expressed as the cycle threshold (Ct) obtained by real-time PCR and normalized to the expression of the GAPDH used as housekeeping gene. Data represent the mean ± sem (standard error of the mean). For each condition, the number of observations is indicated into the brackets. Comparisons of values with those of control (untreated) cells were made with the Dunnet test. Probability p values are reported. Differences are significant when $p < 0.05$.

	WT-ΔNBD2		F508del-ΔNBD2	
	**normalised Ct**	**p**	**normalised Ct**	**p**
Control	1.01 ± 0.07 (5)		0.85 ± 0.20 (4)	
+ VX809	1.18 ± 0.01 (5)	0.83	1.22 ± 0.46 (4)	0.98
+ VX661	1.21 ± 0.01 (4)	0.70	0.88 ± 0.02 (4)	>0.99
+ corr4a	1.22 ± 0.06 (4)	0.47	1.07 ± 0.24 (4)	>0.99
+ VX325	1.00 ± 0.03 (4)	0.28	1.21 ± 0.37 (5)	0.91

Table S6. Evaluation of M1N1-WT protein expression in HEK-t cells whole cell lysates after treatment with correctors and protein new synthesis blockage with cicloheximide. Quantification of protein expression was obtained analysing the band intensity with the software imageJ. Retrieved values were normalized to the intensity of the protein actin used as housekeeper protein. Data represent the mean ± sem (standard error of the mean). For each condition, the number of observations is indicated into the brackets. Comparisons of values with those of control, untreated samples were made with the Dunnet test. Probability p values are reported. Differences are significant when p < 0.05

	**Control**	**+ VX809**	**+VX661**	**+corr4a**	**+VX325**
**0 hour**	162.7 ± 12.7 (4)	233.7 ± 8.2 (5)	201.4 ± 9.6 (5)	144.7 ± 23.0 (4)	169.2 ± 14.3 (4)
		p > 0.99	p = 0.94	p = 0.78	p > 0.99
**1 hour**	139.3 ± 8.8 (4)	225.8 ± 6.5 (4)	189.5 ± 12.4 (5)	124.6± 9.8 (4)	144.1 ± 5.3 (4)
		p = 0.03	p = 0.58	p = 0.89	p = 0.79
**2 hours**	126.6 ± 7.26 (4)	205.7 ± 13.3(4)	178.5 ± 16.2 (4)	113.1 ± 10.1 (4)	133.8 ± 4.0 (4)
		p < 0.01	p = 0.45	p = 0.49	p = 0.68
**4 hours**	109.9 ± 7.7 (4)	154.3 ± 15.9 (5)	132.0± 26.5 (5)	98.7 ± 15.1 (4)	114.4 ± 8.1 (4)
		p < 0.01	p = 0.93	p = 0.12	p > 0.99
**6 hours**	73.4 ± 5.2 (4)	96.1 ± 7.0 (5)	76.4 ± 12.8 (5)	54.9 ± 4605.21 (4)	69.0 ± 0.9 (4)
		p < 0.01	p = 0.63	p = 0.14	p = 0.77
**8 hours**	22.6± 6.3 (4)	41.9 ± 6.0(5)	27.0 ± 8.4 (5)	21.9 ± 1.0 (4)	29.1 ± 2.6 (4)
		p <0 .01	p = 0.93	p = 0.1	p > 0.99

Table S7. Evaluation of M1N1-F508del protein expression in HEK-t cells whole cell lysates after treatment with correctors and protein new synthesis blockage with cicloheximide. Quantification of protein expression was obtained analysing the band intensity with the software imageJ. Retrieved values were normalized to the intensity of the protein actin used as housekeeper protein. Data represent the mean ± sem (standard error of the mean). For each condition, the number of observations is indicated into the brackets. Comparisons of values with those of control, untreated samples were made with the Dunnet test. Probability p values are reported. Differences are significant when $p < 0.05$

	**Control**	**+ VX809**	**+VX661**	**+corr4a**	**+VX325**
**0 hour**	45.7± 2.1 (4)	53.2 ± 9.2 (4)	49.8± 11.0 (4)	53.3 ± 4.3 (4)	58.1 ± 16.6 (4)
		p = 0.89	p > 0.99	p > 0.99	p = 0.57
**1 hour**	41.0 ± 0.5 (4)	5.7 ± 8.38 (4)	54.0 ± 12.3 (4)	46.1 ± 0.4 (4)	53.1 ± 7.6 (4)
		p = 0.95	p > 0.99	p > 0.99	p = 0.61
**2 hours**	33.3 ± 4.0 (4)	68.55 ± 4.5 (4)	67.7 ± 11.1 (4)	33.5 ± 5.4 (4)	45.6 ± 5.7 (4)
		p < 0.01	p < 0.01	p > 0.99	<0.01
**4 hours**	16.9 ± 3.3 (4)	62.6 ± 4.7 (4)	41.3 ± 10.8 (4)	22.5 ± 2.5 (4)	36.2 ± 5.6 (4)
		p < 0.01	p < 0.01	p = 0.14	<0.01
**6 hours**	5.3 ± 3.5 (4)	55.7 ± 4.0 (4)	32.8 ± 8.5 (4)	1.0 ± 0.6 (4)	19.8 ± 4.99 (4)
		p = 0.02	p < 0.01	p = 0.92	<0.01
**8 hours**	0.1 ± 0.03 (4)	14.8 ± 1.6 (4)	10.4 ± 1.4 (4)	0.3 ± 0.1 (4)	4.8 ± 1.7 (4)
		p < 0.001	p < 0.01	p >0 .99	0.5

Table S8. Sequences of forward and reverse primers used to amplify WT- and F508del-M1N1, M2N2, WT- and F508del-CFTR and WT- and F508del-ΔNBD2 constructs and the size expected for each PCR-amplified product. The sequence to amplify the "housekeeping" gene glyceraldehyde-3-phosphate-dehydrogenase (GAPDH) is also shown.

**Target sequence**	**Forward primer**	**Reverse primer**	**Amplicon size (bp)**
WT/F508del-M1N1; WT/F508del-CFTR; WT/F508del-ΔNBD2	5'-GCCAGCGTTGTCTCCAAAC-3'	5'-GCGTTCCTCCTTGTTATCC -3'	327
WT/F508del-M2N2	5'-GAACAGTTTCCTGGGAAGC-3'	5'-CTAAAGCCTTGTATCTTGC-3'	459
GAPDH	5'-CAAGGTCATCCATGACAACTTTG-3'	5'-GTCCACCACCCTGTTGCTGTAG-3'	496

Reaction mixtures were processed in a CFX Connect Real-Time PCR Detection System instrument (Bio-Rad Laboratories, Hercules, CA, USA). The PCR reaction mixture contained 1.5 μM of the appropriate forward and reverse PCR primers (Bio-Fab Research, Roma, Italy), 1.5 U of SsoAdvance Universal SYBR Green Supermix (Bio-Rad Laboratories) and 100 ng cDNA in 20 µl. The PCR protocol consisted of an initial denaturation at 95 °C for 30 s, followed by 35 cycles of a denaturation step at 95 °C for 10 s, an annealing step at 54 or 56 °C for 30 s and an extension step at 72 °C for 50 s. Appropriate negative and positive controls were included. A melting curve analysis was used to evaluate the presence of secondary non-specific products.

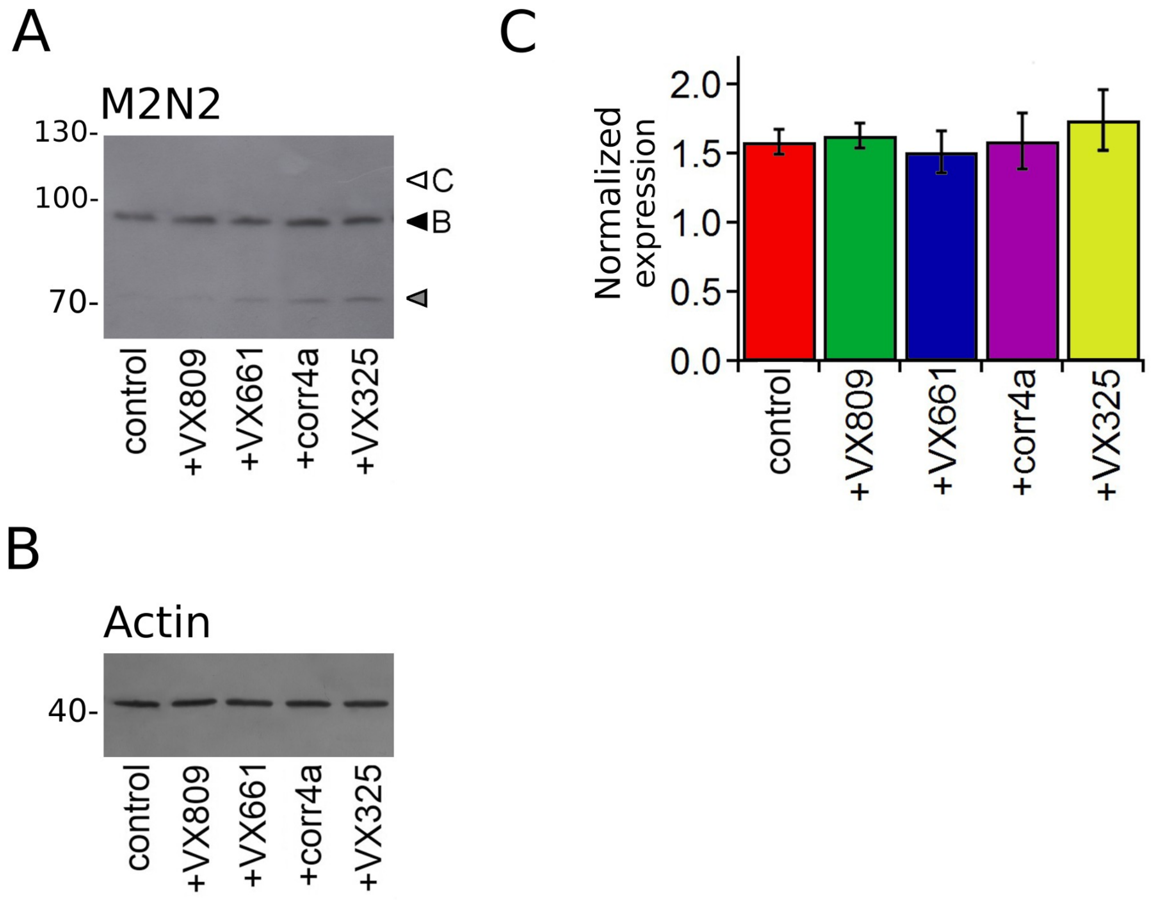

Figure S1. Expression of M2N2 polypeptide. (**A**) Western blot of lysates of M2N2 transiently transfected HEK-t cells treated with DMSO (control), VX809, VX661, corr4a and VX325 is shown on the left. White, black and grey arrowheads indicate the mature, fully glycosylated (band C), immature core-glycosylated (band B) and, probably, the unglycosylated forms of the protein. (**B**) Expression of the housekeeper protein actin in the same samples of (**A**). The molecular weight of the proteins of the molecular weight marker that was run in the SDS-PAGE is indicated on the left of each blot. (**C**) Bar graph indicating the relative quantification of the M2N2 protein in the 5 examined conditions. Data are expressed as mean ± standard error of the mean (sem) of at least 4 independent experiments. Statistical comparison of the data was made by Dunnet multiple comparison test (all groups against control group). Asterisks indicate a statistical significance versus control: *$p < 0.05$.
